# The isolated orbital floor fracture from a transconjunctival or subciliary perspective-A standardized anthropometric evaluation

**DOI:** 10.4317/medoral.20818

**Published:** 2015-11-22

**Authors:** Gregor Raschke, Gabriel Djedovic, Andre Peisker, Rene Wohlrath, Ulrich Rieger, Arndt Guentsch, Marta Gomez-Dammeier, Stefan Schultze-Mosgau

**Affiliations:** 1 MD, DDS, PhD. MD, DDS. DMD. Department of Plastic Surgery & Cranio-Maxillofacial Surgery, Friedrich Schiller University Jena, Erlanger Allee 101, Jena, Germany; 2 MD. Department of Plastic, Reconstructive & Hand Surgery, Innsbruck Medical University, Anichstrasse 35, Innsbruck, Austria; 3 MD, PhD. Department of Plastic & Aesthetic, Reconstructive & Hand Surgery, St. Markus Hospital, Johann Wolfgang von Goethe University, Frankfurt/Main, Germany; 4 DMD, PhD. Marquette University School of Dentistry, 250 W Wisconsin Ave., Milwaukee, WI 53233, United States of America

## Abstract

**Background:**

The influence of orbital fractures and their repair on the rate of deformities of the lower eyelid is an ongoing source of discussion in the literature. Most of the present studies include isolated blowout as well as combined orbital fractures.

**Material and Methods:**

We present a retrospective evaluation of a series of 100 patients after isolated blowout fracture repair using reference anthropometric data on standardized photographs. Analysis included eye fissure width and height, lid sulcus height, upper lid height, upper and lower iris coverage, position of cornea to palpebra inferior, canthal tilt, scleral show, ectropion and entropion. It was clearly distinguished between operated and contralateral eyelid, whether a transconjunctival or a subciliary approach was performed and amount of fracture. Our main interests were changes of the aforementioned parameters with regards to eyelid deformities.

**Results:**

Surgery per se did not significantly influence eyelid deformities. However, the surgical approach selected significantly affected eye fissure index, lower iris coverage and rate of scleral show, indicating retraction of the lower eyelid.

**Conclusions:**

The standardized measurements described here are accurate and objective to evaluate postoperative results. The subciliary approach included the highest risk of lower lid retraction as compared to transconjunctival approaches.

**Key words:**Transconjunctical approach, subciliary approach, orbital floor fracture.

## Introduction

According to our experience, before undergoing surgical repair of a blow out fracture, most patients worry about the risk of distortion of the face and especially the eyelids. Even minimally displaced blowout fractures may result in aesthetic and functional deformities of the periorbital region (1).

There is an ongoing discussion in the literature about the optimal treatment of orbital floor fractures. Especially the discussion about how to approach the orbital floor is ongoing. To date most studies comparing transconjunctival and transcutaneous approaches include patients with isolated orbital floor fractures, zygomaticomaxillary fractures and combined orbitomaxillary fractures altogether (2,3) without giving results clearly distinguishing between these different entities of fractures. It seems reasonable, as reported earlier, that different severity and type of trauma have significant impact on the risk of developing an en-or ectropion (3). Thus the inclusion of different types of fractures of the orbita in studies referrring and/or comparing transcutaneous and transconjunctival approaches limits their validity.

Only few articles referring to a single type of fracture are available. These articles mostly report on the outcome of isolated blowout fractures (4-7). They report the clinical management (7), functional outcome (5,6) and clinical outcome of the surgical method (4,6). There is a lack of elaborated and objective assessments of the effect of blow out fractures and its surgical treatment on the eyelid architecture in the current literature.

However, such an assessment is highly desirable, as it may help to quantify the influence of trauma and particular surgical procedure selected on the eyelid morphology. Normative anthropometric measurements of the face are available (8-13). Their benefit in planning, performance and evaluation of facial surgery is widely recognized (11,12,14). In a group of 100 patients suffering from isolated blowout fractures, anthropometric measurements were performed on standardized photographs. We investigated differences between the affected and the contra lateral side and either a transconjunctival or a subciliary approach was performed. Furthermore we evaluated the influence of the type of orbital floor fracture.

## Material and Methods

Before the study was initiated, the local Ethics Committee of the University Hospital Jena was asked to give his approval to the study. Because the study design aimed to evaluate routinely performed documentation like standardized photographies or X-rays and did not influence the the diagnostical or therapeutic process the Ethics Committee denied the necessity of special ethical approval. Prior to surgery all included patients signed an informed consent permitting the scientific evaluation of their routinely recorded documentation including x-rays and photographies.

All patients were operated at the Department of Plastic Surgery & Cranio-Maxillofacial Surgery at the University Hospital Jena, Germany, between January 2006 and December 2011. The inferior orbital rim and orbital floor were exposed either through a subciliary or a transconjunctival approach, which were performed in a standardized manner.

**Figure 1:**
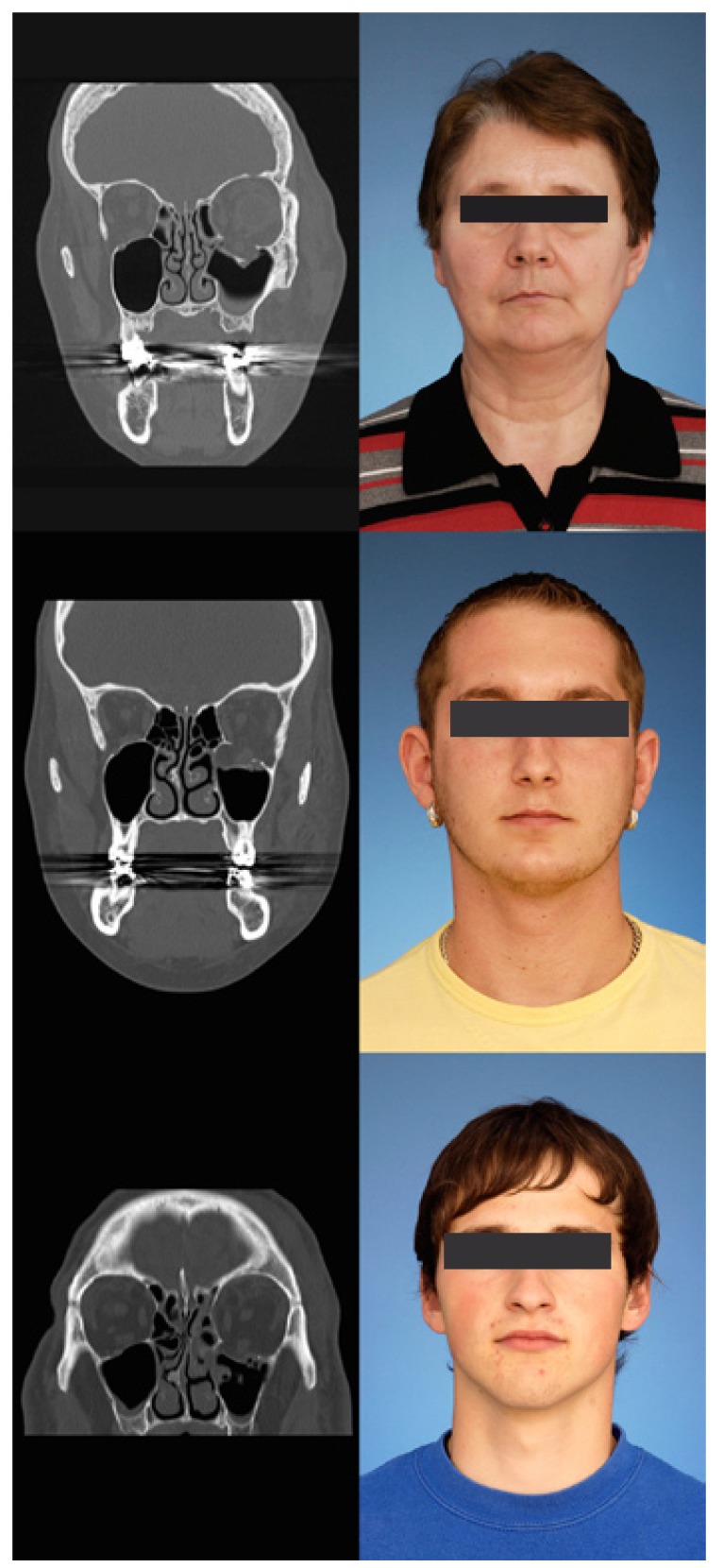
On the left coronar CT scan, on the right standardized photography three months after surgery. The patients above and in the middle were operated through a transconjunctival approach, the patient below through a subciliary approach.

The subciliary approach was performed in the manner of a step dissection, the transconjunctival approach in a retroseptal technique. A photo- and radiographic description of three patients is shown in figure 1.

Colored frontal view photographs with open eyes were taken postoperatively, after definite wound healing, with a Nikon D 80 camera (objective: Nikon AF Micro Nikkor 105 mm 1:2.8 D; aperture: f13; Nikon Corp, Tokyo, Japan) with a standardized lens at a patient distance of 1 m in a standardized position and a slit lamp by a professional photographer. Only photographs in which the inter pupillary axis was at the same level as the camera lens and faces were clearly at rest were selected to minimize photographic distortion (15,16). Further analysis was performed using Adobe Photoshop CS2 (Adobe Inc, San Jose, CA).

**Figure 2:**
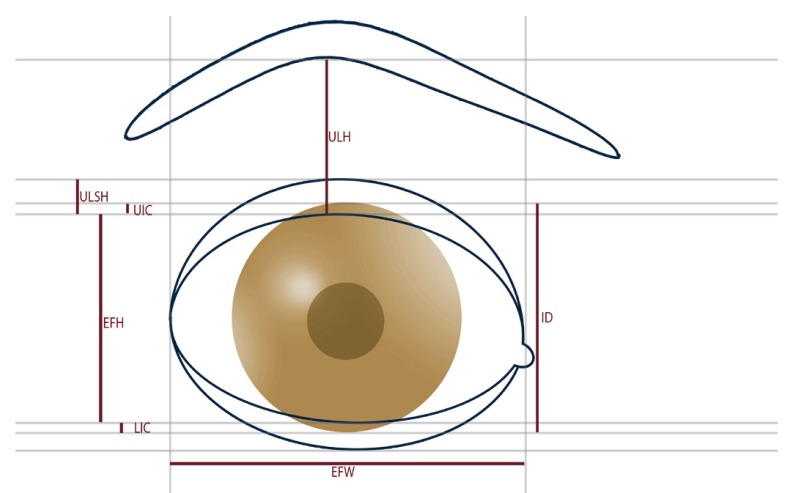
Schematic picture with description of the used anthropometric distances ULSH indicates upper lid sulcus height; ULH, upper lid height; UIC, upper iris coverage; LIC, lower iris coverage; ID, iris diameter; EFH, eye fissure height; EFW, eye fissure width.

**Figure 3:**
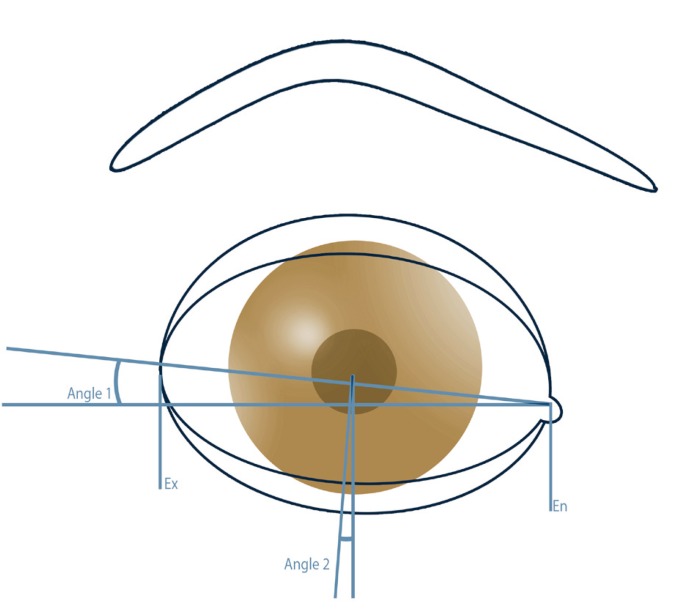
Schematic picture of canthal tilt (An1), describing the inclination of the horizontal axis of the eye between endocanthion (En) and exocanthion (Ex). Furthermore description of the position of the lower iris (An2) as the aberration of the contact point between cornea and lower eyelid from the vertical reference line through the center of the iris.

On the basis of predefined landmarks and data ( Table 1), the following anthropometric dimensions based on the work of Farkas and Munro (9-12,14) as well as well known clinical data were investigated (Fig. 2): Eye Fissure Index is defined by the eye fissure height (EFH, Ps-Pi), the vertical distance from the margin of the inferior palpebra to the margin of the superior palpebra. The EFH was then divided by the eye fissure width (EFW, en-ex), which is defined by the intercanthal distance. The eyelid sulcus of the upper eyelid divides the upper eyelid in an upper and lower part. The upper lid sulcus height (ULSH, LS-Ps) is depicted by the vertical distance between the upper palpebral margin and eyelid sulcus. as percentage of the upper lid height (ULH, Os-Ps), the distance between orbitale superioris and upper palpebral margin. Upper iris coverage (UIC) represents the part of the upper iris covered by the upper eyelid. It was investigated by halving iris diameter and subtracting the free visible upper radius of the iris (Ic-Ps) as percentage of the total iris diameter (ID). Lower iris coverage (LIC) represents the part of the lower iris covered by the lower eyelid. It was raised by halving the iris diameter and subtracting the free visible upper radius of the iris (Ic-Pi). In the case of scleral show or ectropion its values turned negative. The position of the lower eyelid to the lower iris describes the angulation of the inferior eyelid to the center of the iris (8). It was measured by placing a vertical reference line through the center of the iris (Ic). Another line was drawn through the center of the iris (Ic) and the point of contact of the lower eyelid and cornea (Ic-CPi). The angle formed by both lines was measured in degrees (Fig. 3). Medial deviations of the angle were measured as negative, lateral deviations as positive value. Canthal tilt describes the intercanthal fissure inclination (13) measured as the angle between the EFW (en-ex) and a horizontal reference line passing through the endocanthion in degrees (Fig. 3). Furthermore the rate of scleral show, ectropion, and entropion was recorded.

**Table 1:**
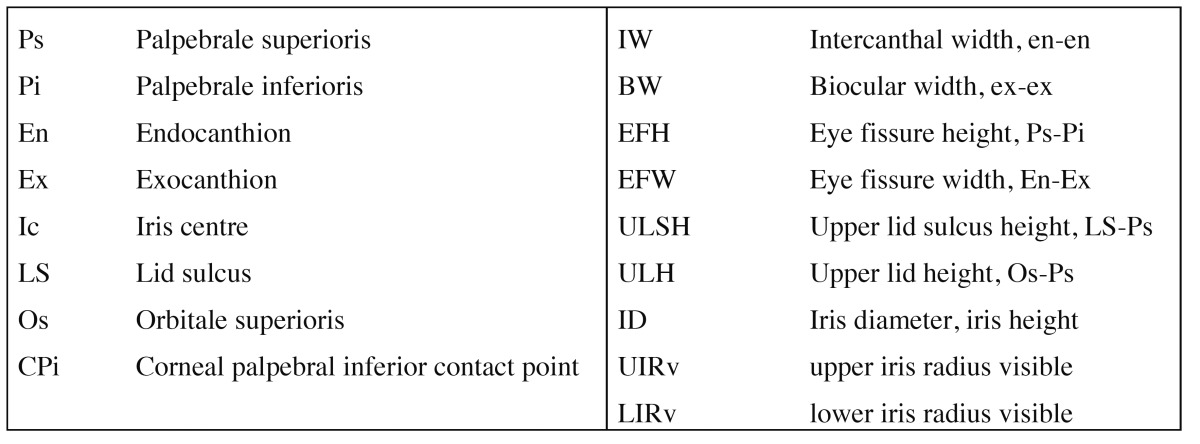
Used anthropometric landmarks and distances based on the investigations by Farkas.

All parameters were measured on both eyes. Results were evaluated comparing the operated and the contra lateral (not operated, control) side. The impact of whether a transconjunctival or a subciliary approach was performed was evaluated, as well. Furthermore the influence of the type of orbital floor fracture was investigated through an analysis of operation reports and preoperative CT scans with coronal and sagittal reformations. Type 1 consisted of small fractures of the anterior medial orbital floor and type 2 of larger fractures involving the orbital floor and medial wall (17). Occurrence of diplopia was extracted out of patients´ records.

In order to analyse the influence of operated and contralateral side, surgical approach selected and type of orbital floor fracture on EFI, ULSH, UIC, LIC, position of lower eyelid to lower iris and canthal tilt, univariate and mixed model ( Table 2) ANOVAs were conducted. Fisher’s Exact Tests were conducted to compare operated and contralateral eyes with reference to ectropion and scleral show. All calculations were done using SPSS V 19.0 for Windows (SPSS, Inc, Chicago, IL).

**Table 2:**
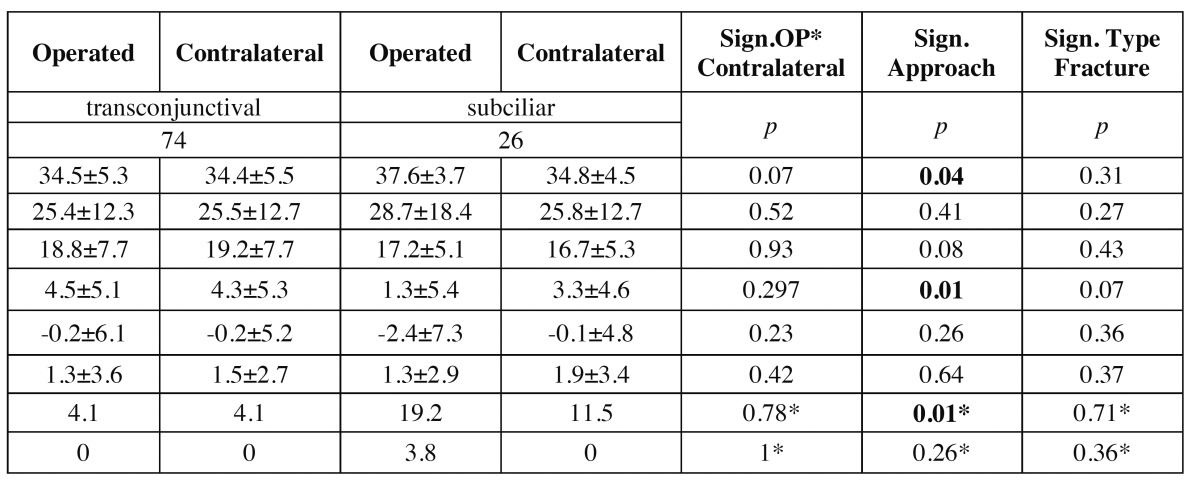
Comparison of the results of the photographic measurements of operated and the contralateral eyelids, surgical approach selected and fracture type.

## Results

All patients included suffered from a unilateral isolated blowout fracture. 90 white Caucasian patients, 72 men (72.0%) and 28 women (28.0%) were operated. Average age was 42.08±18.70 at time of surgery. Reconstruction of the orbital floor was performed in 64 patients (64.0%) by a polydioxanone sheet, in 32 patients (32.0%) by a titanium mesh. Three patients (3.0%) did not need alloplastic reconstruction of the orbital floor. The orbital floor was exposed via a transconjunctival approach in 74 cases (74.0%), 52 men (70.3%) and 22 women (29.7%), and via a subciliary approach in 26 cases (26.0%), 20 men (76.9%) and 6 women (23.1%). In 62 cases (62.0%), 44 men (71.0%) and 18 women (29.0%), a Type 1 Fracture was observed, in 38 cases (38.0%), 28 men (73.7%) and 10 women (26.3%), a Type 2 fracture. No entropion was observed.

The postoperative photographs evaluated were taken 3 months after surgery. A comparison of the results of the photographic measurements differentiated between operated and the contra lateral eyelid, surgical approach and type of fracture is shown in  Table 2. None of the investigated parameters presented a significant difference between operated and contra lateral side.

The surgical approach to the orbital floor significantly influenced EFI (*p*=.04), LIC (*p*=.01) and the rate of scleral show (*p*=.01). The other investigated parameters presented no significant correlations with the surgical approach selected. One ectropion was observed in the group of a subciliary approach.

The type of orbital floor fracture did not significantly influence on the parameters investigated.

The multivariate analysis performed did not yield significant interaction effects between the factors operated or not, surgical approach and type and severity of fracture. However, for statistical reasons a significant interaction effect is not required to confirm the significant effect of the surgical approach on EFI, LIC and scleral show values.

Two patients (2.2%) suffered from persistent diplopia in the direction of ocular elevation at the time the postoperative photographs were taken. Both patients underwent a transconjunctival approach. None of them presented symptoms of entrapment or enophtalmos in the postoperative ophthalmologic examination. We were unable to find a medical, anatomic or surgical reason, which is not unusual (15).

## Discussion

A blowout fracture is defined as a fracture of the orbital floor. It does not involve the orbital rim. Besides the description of functional disabilities the most common criteria of postoperative evaluation of orbital floor fracture repair consists in the rate of lower lid retraction, ectropion and entropion (4). These common criteria do not allow detection of more subtle changes of the periorbital architecture.

The presented anthropometric measurements of the periorbital region may help us to objectify the morphologic outcome of orbital floor fracture repair. As different grades of severity and types of trauma play a decisive role in the risk of development of en- or ectropion (2,3), we included only isolated blowout fractures in our study, to improve the validity of our data. The significance of the investigation of the impact of subciliary or transconjunctival approaches on the periorbital architecture are enhanced thereby, as well.

Orbital floor fractures result from an abrupt increase of intraorbital pressure and may be caused either by direct contact to the globe or contact with the inferior orbital rim causing the floor to buckle. Forces applied to the orbital rim, described by Waterhouse *et al*. as type 1, rather lead to small fractures of the mid medial floor and rarely herniation of orbital content. Forces applied to the globe rather lead to larger fractures including the orbital floor and medial wall and herniation of orbital content and were described by Waterhouse *et al*. as type 2 (17). Due to the potential influence of the type of fracture to postoperative eyelid malposition this easy and reproducible classification was used to investigate the influence of amount of fracture on eyelid morphology.

Several anthropometric measurements of the periorbital region have been described (9,10,12,14). We used the eye fissure index (EFI), upper lid sulcus height (ULSH), upper (UIC) and lower (LIC) iris coverage, canthal tilt and position of lower eyelid to iris in our study.

The eye fissure width, measured between the endo- and exocanthion, is referred to equal 30 mm. The eye fissure height between the margins of the upper and lower palpebra is reported to be 9-10 mm with open eyes straight ahead (18). Because linear measurements are not exactly reproducible in standardized photographs, we preferred to apply the EFI reflecting the relation between EFH and EFW.

The LIC is very important for the look of the patient. The normative value is 7% (12). Negative values occur in the case of scleral show. Sclera should normally not be visible looking straight ahead (8). A reproducible photographic quantification of scleral show is desirable for the judgment of the quantity of distortion. Therefore scleral show was quantified by changes of EFI and LIC.

Ectropia are linked to lower lid retraction, as well, but not in such a direct manner as scleral show. Scleral show describes a general and symmetric decline of the lower eyelid attached to the eyeglobe. In case of an ectropion the lower eyelid turns inside out, leaving the inner eyelid and globe surface exposed and is subsequently prone to irritation. It may occur medially or laterally or on both sides and does not inevitably go along with excessive lower lid retraction.

Measurements of the upper eyelid position were included in our study in order to secure that changes of the morphology of the upper eyelid did not affect the measurements of EFI. ULSH is a helpful measurement in the appraisal of the composition of the eyelid to the eyebrow. UIC reflects the covered part of the upper iris (12).

To adequately describe the shape of the eyelids two angles exhibiting decisive impact on the periorbital appearance were measured: Canthal tilt (13) is of big concern for the facial appearance. Sad look may be the consequence of a negative canthal tilt (8). It was referred to be 2 mm or at an angle of 10 to 15 degrees above the medial canthus (19).

The position of lower eyelid relative to iris describes the normal contact point of the lower palpebra to the limbus corneae at the 6 o´clock position (8).

Clearly identifiable eyelid distortions such as unilateral lower lid retraction and scleral show or a lowered canthal tilt lead to an unpleasant appearance, which often is noticed by the patients themselves.

Altogether the nine presented anthropometric and clinically relevant parameters described in this study are able to describe and quantify such malpositions. They were easily and reproduciblely definable in the frontal view photographs and may be influenced by a blowout fracture or its surgical repair. The comparison of postoperative photographs by surgeons and/or independent observers seems less reproducible to us than the presented anthropometric measurements.

The consideration of the anthropometric parameters described may be relevant not only for scientific purposes but also in the clinical care of these patients. If in the further clinical course a surgical revision is warranted, it is important to exactly plan the degree of correction necessary. In order to achieve the best result possible it is not only necessary to exactly estimate the degree of vertical correction described in this study by EFI and LIC, but also to achieve an appealing shape of the lower eyelid towards the globe. Canthal tilt and position of lower eyelid to iris may facilitate this estimation.

In the presented study we aimed to focus on morphologic aspects and the influence of trauma and surgical approach. Previous studies indicated, that the interpretation of the raw data of ophthalmologic findings do not correlate with the “real life” rate of complications. Therefore the ophthalmologic evaluation has to be interpreted for every individual patient and was not evaluated and discussed in detail in this current study (5).

The comparison of operated and contralateral side as well as of the surgical approach to the orbital floor did not exhibit a significant effect on ULSH, UIC, canthal tilt and position of lower eyelid to iris (see  Table 2). The constant values of UIC and ULSH indicate that, not surprisingly, the architecture of the upper eyelid and the shape of the eyelids were not influenced by the blowout fracture and its subsequent repair.

EFI and LIC did not show significant differences, when operated and contralateral side were compared (see  Table 2). This underlines, that preexisting scleral show on one side, which is often associated with scleral show on the contralateral side, has no significant influence on the rate of postoperative scleral show. Furthermore it could be interpreted as an indication, that surgery itself is not associated with higher rates and amount of eyelid deformities.

However increased values of EFI, decreased values of LIC and an increased rate of sleral show were observed when a subciliary approach was performed. This indicates lower lid retraction, which did not seem to occur in a significant manner, when a transconjunctival approach was performed (see  Table 2).

In this study one ectropion was observed. This may be related to the lower number of patients included in this study undergoing a subciliary approach. In previous studies similar or even lower rates of ectropion were observed. Overall these results are endorsed by the present literature: Lower eyelid retraction is the most common complication after a subciliary approach (20,21). Scar contracture, cicatricial connection between the septum orbitale, orbicularis muscle and surrounding tissue as well as loss of muscle tonus may provoke scleral show and ectropion. Thus most authors prefer the transconjunctival approach (4,6,15,20,22-24). Transconjunctival approaches reduce complications such as ectropion to a minimum (2), but include the highest risk of entropion (3).

During the past decades the transconjunctival approach showed an uninterrupted increasing use. Altogether transconjunctival incisions seem to include a lower risk of postoperative lower lid retraction and ectropion compared to transcutaneous and especially subciliary approaches, as suggest our data (see  Table 2).

The classification of orbital floor fractures investigated here did not yield significant influence on the eyelid morphology in our study. Previous analyses investigating other classifications of orbital floor fracture localizations reconfirm this result (4). Altogether this may be interpreted as evidence, that a postoperative lower eyelid malposition is more dependent on the selection of the surgical approach than on the localization and type of the fracture.

In our center we prefer the transconjunctival approach whenever possible. To our experience, the rate of ecor entropion is related to inexperience. The level of the incision in the fornix is enormously relevant. The preservation of the septal integrity as provided by the retroseptal incision seems most likely to us to prevent lower eyelid distortion (15).

We do not see indications for a transcutaneous approach in isolated blow-out fractures, which are all satisfactorily access able through a transconjunctival approach.

Only in case of more-fragment-fractures of the inferior or lateroinferior orbital rim requiring extensive exposure we do see indications for a transcutaneous approach in the form of a subtarsal approach. The incision of the subtarsal approach should be placed as close as possible to the inferior border of the tarsal plate.The subtarsal approach was judged to be cosmetically acceptable when concealed within a rhytid and less risky in matters of lid retraction than subciliary approaches (20-22,25-28).

## Conclusion

Analyses of orbital fractures repair results should clearly distinguish isolated and combined orbital floor fractures. The evaluation of the effects of isolated blowout fractures and their operative therapy on the periorbital architecture by using anthropometric data extracted from standardized photographs is reliable and adequate. The subciliary approach exhibited a significantly higher rate of lower lid retraction than the transconjunctival approach.
